# Protocol for standardized minimally invasive mouse models of bisphosphonate-related and radiation-induced jaw osteonecrosis

**DOI:** 10.1016/j.xpro.2026.104851

**Published:** 2026-10-05

**Authors:** Zhangfan Ding, Jiayuan Zhang, Hanyu Liu, Abhishek Chandra, Makarand V. Risbud, Anjali P. Kusumbe, Junyu Chen

**Affiliations:** 1 State Key Laboratory of Oral Diseases, National Center for Stomatology, National Clinical Research Center for Oral Diseases, West China Hospital of Stomatology, Sichuan University, Chengdu 610041, China; 2 Tissue and Tumor Microenvironments Lab, Cancer Discovery and Regenerative Medicine Program, Lee Kong Chian School of Medicine, Nanyang Technological University, Singapore 636921, Singapore; 3 Multidisciplinary Institute of Ageing (MIA-Portugal), University of Coimbra, 3004-504 Coimbra, Portugal; 4 Department of Physiology and Biomedical Engineering, Mayo Clinic, Rochester, MN 55905, USA; 5 Robert and Arlene Kogod Center on Aging, Mayo Clinic, Rochester, MN 55905, USA; 6 Department of Biochemistry and Molecular Biology, Mayo Clinic, Rochester, MN 55905, USA; 7 Department of Orthopaedic Surgery, Sidney Kimmel Medical College, Thomas Jefferson University, Philadelphia, PA, USA; 8 Graduate Program in Cell Biology and Regenerative Medicine, Thomas Jefferson University, Philadelphia, PA, USA

**Keywords:** Health Sciences, Model Organisms, Biotechnology and bioengineering

## Abstract

We present a protocol for establishing murine models of bisphosphonate-related osteonecrosis of the jaw (BRONJ) and osteoradionecrosis of the jaw (ORNJ). We describe steps for a uniform, low-trauma tooth extraction technique with disease-specific induction, systemic zoledronic acid for BRONJ and precision-targeted irradiation for ORNJ, to generate robust and clinically relevant models of jaw osteonecrosis, approaching minimizes procedural variability and off-target effects, yielding robust, clinically relevant phenotypes. This protocol provides a standard and minimally invasive framework for investigating jaw osteonecrosis arising from distinct etiologies.

## Highlights

• A platform for bisphosphonate-related and radiation-induced osteonecrosis in mouse jaws
• Steps for mandibular molar extraction using bent micro-forceps for less trauma
• Procedures for mandible-targeted irradiation minimizing off-target radiation injury
• A unified experimental framework for multimodal analysis of jaw osteonecrosis

## Abstract (editor highlights)

Publisher’s note: Undertaking any experimental protocol requires adherence to local institutional guidelines for laboratory safety and ethics.

## Graphical abstract

**Figure:**
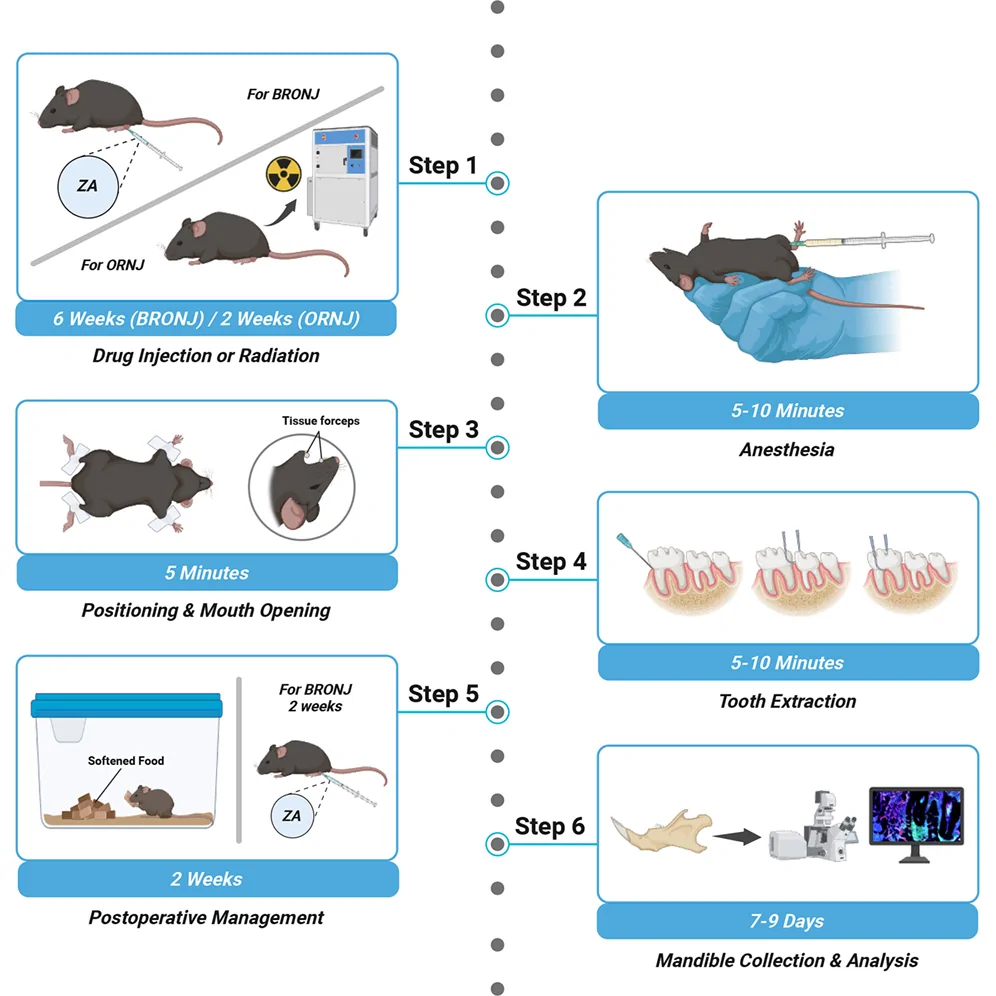

## Before you begin

Osteonecrosis of the jaw (ONJ) represents a severe and debilitating complication associated with widely used clinical therapies. It primarily includes two major clinical subtypes: bisphosphonate-related osteonecrosis of the jaw (BRONJ) and osteoradionecrosis of the jaw (ORNJ).

Bisphosphonates are widely used in the treatment of skeletal disorders, including osteoporosis and multiple myeloma,^1^^,^^2^ while radiotherapy remains a cornerstone in the management of head and neck malignancies.^3^^,^^4^^,^^5^ Despite their clinical benefits, both therapies are associated with severe late complications in the maxillofacial region, collectively manifesting as jaw osteonecrosis. Bisphosphonate-related osteonecrosis of the jaw (BRONJ) and osteoradionecrosis of the jaw (ORNJ) are clinically distinct but pathophysiologically related conditions characterized by progressive bone destruction, chronic infection, fistula formation, and pathological fracture, which severely impair oral function and quality of life.^3^^,^^6^

Although these conditions are increasingly recognized, their underlying mechanisms remain incompletely understood, and effective disease-modifying therapies are still lacking. A major limitation in the field is the absence of standardized and reproducible preclinical models that reliably recapitulate key pathological features across different etiologies. Existing experimental approaches are often heterogeneous and technically demanding, leading to substantial variability and limited cross-study comparability.^4^^,^^7^^,^^8^^,^^9^

To address this gap, we describe standardized experimental protocols for establishing minimally invasive mouse models of BRONJ and osteoradionecrosis of the jaw (ORNJ). These protocols are designed to improve procedural consistency and reproducibility while reducing technical variability. Mice in the BRONJ group received intraperitoneal ZA injections (200 μg/kg) three times weekly for eight weeks. This regimen was selected to model the cumulative skeletal exposure associated with clinical BRONJ development rather than the intermittent annual 5 mg intravenous ZA regimen commonly used for osteoporosis treatment. Clinical BRONJ is typically associated with prolonged bisphosphonate exposure, with a reported median cumulative ZA exposure of approximately 126 mg over 36 months (∼42 mg/year).^10^ Based on FDA-recommended body surface area conversion, this corresponds to a human-equivalent exposure of approximately 16 μg/kg, or ∼49 μg/kg weekly.^11^ Frequent three times weekly dosing is pharmacokinetically necessary because over 60% of intravenous ZA binds irreversibly to bone with a multi year half life.^12^ Infrequent dosing cannot achieve pathological bone accumulation in mice, while three times weekly dosing ensures steady saturation. This dose fits the established murine BRONJ range of about 100–300 μg/kg per injection for BRONJ models.^13^ The models support downstream applications including micro-CT imaging, histological assessment, and molecular analyses, and provide a robust experimental framework for studying the mechanisms and therapeutic interventions of jaw osteonecrosis.

Based on validated effect sizes in murine BRONJ/ORNJ models,^13^^,^^14^ we recommend n=8 mice per group for micro-CT and histology endpoints, and n=10 per group for immunofluorescence, to achieve >80% power while accommodating perioperative attrition (estimated ≤20%). For pilot studies, n=6 may suffice if paired with within-mouse contralateral controls.

### Institutional permissions

All the animals utilized for the studies were housed at Sichuan University. All animal procedures were reviewed and approved by the Research Ethics Committee of West China Hospital of Stomatology (Approval Nos. WCHSIRB-D-2023-245, WCHSIRB-D-2024-248). Adult male C57BL/6 mice were obtained from the Charles River Laboratories (USA). Mandatory postoperative analgesia (buprenorphine 0.05–0.1 mg/kg SC every 8–12 h for 72 h) is included per IACUC approval.

### Innovation

This protocol establishes a standardized and reproducible minimally invasive approach for generating mouse models of bisphosphonate-related osteonecrosis of the jaw (BRONJ) and osteoradionecrosis of the jaw (ORNJ), improving procedural consistency and reducing inter-operator variability. A unified oral surgical strategy is applied across both models, integrating tape-assisted mouth opening and blunt tissue retraction to enable stable and atraumatic exposure of the mandibular molar region. In BRONJ, systemic zoledronic acid administration is combined with controlled mandibular first molar extraction using bent micro-forceps to achieve precise and reproducible tooth removal. In ORNJ, a customized lead shielding system enables noninvasive, mandible-targeted irradiation with improved spatial precision and reduced off-target effects. Together, these refinements provide a robust and reproducible framework for downstream micro-CT, histological, and immunofluorescence analyses and for mechanistic studies of jaw osteonecrosis and therapeutic evaluation.

## Key resources table

**Table:** 

REAGENT or RESOURCE	SOURCE	IDENTIFIER
**Antibodies**		

CD31 (1:200)	Abcam	Cat# ab24590; RRID:AB_448167
Endomucin (1:200)	Santa Cruz Biotechnology	Cat# sc-65495; RRID:AB_2100037
Sp7 (1:200)	Abcam	Cat# ab209484; RRID:AB_2892207
Donkey anti-Mouse IgG (H+L) Alexa Fluor 488 (1:500)	Invitrogen	Cat# A-21202; RRID:AB_141607
Donkey anti-Rat IgG (H+L) Alexa Fluor Plus 555 (1:500)	Invitrogen	Cat# A48270; RRID:AB_2896336
Donkey anti-Rabbit IgG (H+L) Alexa Fluor 647 (1:500)	Invitrogen	Cat# A-31573; RRID:AB_2536183

**Chemicals, peptides, and recombinant proteins**		

Zoledronic acid (ZA)	APExBIO	Cat# A1352
NaCl	Sigma	Cat# S7653
Tribromoethyl alcohol (Avertin)	Biotopped	Cat# Top9153S
Iodine (povidone-iodine)	Sigma-Aldrich	Cat# PVP-I
Ethanol	Sigma-Aldrich	Cat# 1070172511
Sodium hyaluronate eye drops	Qilu Pharmaceutical	N/A
PBS (10×)	VWR	Cat# 437117K
Donkey serum	Solarbio	Cat# SL050
Paraformaldehyde (PFA)	Sigma-Aldrich	Cat# P6148
EDTA	Sigma-Aldrich	Cat# E6758
Polyvinylpyrrolidone	Sigma-Aldrich	Cat# P5288
Sucrose	Sigma-Aldrich	Cat# V900116
Gelatin	Sigma-Aldrich	Cat# G1890
Fluoromount-G	Invitrogen	Cat# 00-4958-02
Triton X-100	Sigma-Aldrich	Cat# T8787
H&E staining kit	Servicebio	Cat# G1005-100ML
DAPI	Sigma-Aldrich	Cat# D9542

**Experimental models: Organisms/strains**		

Mouse: wild-type C57BL/6J, 8- to 12-weeks-old male	Charles River Laboratories (USA)	Strain#632

**Software and algorithms**		

Dataviewer (version 1.5.6.2)	Bruker	N/A
CTVox (version 1.18.4.0)	Bruker	N/A
OlyVIA (version 4.1)	Olympus/Evident Scientific	N/A
Imaris (version 10.1.0)	Bitplane	N/A
Imaris File converter (version 10.1.0)	Bitplane	N/A
Adobe Illustrator CC	Adobe	N/A
X-ray irradiator	Rad Source Technologies	RS 2000Pro
Micro-CT scanner	SCANCO	μCT45
Cryostat	Leica	CM1950
Digital slide scanner	Olympus	SLIDEVIEW VS200
Spinning disk confocal microscope	Olympus	SpinSR

**Other**		

Ophthalmic scissors (straight, 10 cm)	Biosharp	Cat# BS-SOR-S-100
Micro-forceps (straight, 14 cm)	Biosharp	Cat# BS-ZER-S-014A
Blunt tissue forceps	Biosharp	Cat# BS-ZER-S-100
Animal bowl	Servicebio	Cat# QX0030
Low-profile blades	Leica	Cat# 14035838382
Cover glass	VWR	Cat# 631-0135
Heating pad	N/A	N/A
Focusable Yellow LED Lamp	N/A	N/A

**CRITICAL:** PFA is toxic. The fixative solution should be prepared inside a fume hood.

## Materials and equipment

### Injection solution

Dissolve ZA powder in 8 mL 0.9% NaCl solution (normal saline).

**CRITICAL:** This solution should be stored at 4°C for a maximum of 1 month.

**Table:** Fixation solution

REAGENT	Final Concentration	Amount
Paraformaldehyde	4%	4 g
PBS (1×)	1×	100 mL

Store at 4 °C for up to 1 weeks. Prepare fresh for best results.

**CRITICAL:** PFA is highly toxic and should be manipulated in the fume hood, and the appropriate PPE is required.

### Decalcifying solution (0.5 M EDTA, pH 7.4)

Weigh 14.61 g EDTA into 80 mL distilled water. While stirring, add NaOH pellets (or concentrated NaOH solution) gradually to dissolve the EDTA and simultaneously adjust pH to 7.4. Bring final volume to 100 mL with distilled water. Filter if slight turbidity remains.

**CRITICAL:** EDTA free acid does not fully dissolve without alkalinization. And pH adjustment and dissolution proceed together. Store at 20–25°C for up to 1 month. For immunofluorescence applications, decalcification is performed at 4°C. Based on our established protocols, durations are strictly controlled: 24–36 h for young mice (<3 months) and 48 h for aged mice (>18 months).^15^^,^^16^ This minimizes antigenic loss while rendering the tissue suitable for sectioning. For routine HE staining requiring complete demineralization, decalcification is extended and validated by X-ray radiography.

**Table:** Antibody solution

REAGENT	Final Concentration	Amount
Donkey serum	5%	50 μL
Antibodies	1:200 or 1:500	2 or 5 μL
PBS	1×	945 or 948 μL

**Table:** Antibody dilutions

Antibody	Dilution
CD31	1:200
Endomucin	1:200
Sp7	1:200
Donkey anti-Mouse IgG Alexa Fluor 488	1:500
Donkey anti-Goat IgG Alexa Fluor 555	1:500
Donkey anti-Rabbit IgG Alexa Fluor 647	1:500

**CRITICAL:** The antibody solution should be stored at 4°C, protected from light, for a maximum of 1 week. Prepare fresh for best results.

## Step-by-step method details

### Preoperative management

**Timing: 6 weeks for BRONJ or 2 weeks for ORNJ**

This section describes the preoperative procedures for BRONJ and ORNJ, followed by tooth extraction, postoperative care, and downstream analyses. Preoperative management is divided into Part 1 for BRONJ and Part 2 for ORNJ.

1. For BRONJ: Preoperative drug injection
  **Timing: 6 weeks**
  ***Note:*** This section describes the process of drug injection into mice for the model of mandibular bone necrosis.
  a. House mice (8–12 weeks old male C57BL/6J) in standard cages with a 12 h light/dark cycle and ad libitum access to food and water.
    ***Note:*** Only male mice are used to standardize bone metabolic status and eliminate hormonal variability associated with the estrous cycle.
  b. Weighing each mouse. Inject the mice intraperitoneally with the prepared solution at a dose of 200 μg/kg of ZA, 3 times a week.
    **CRITICAL:** This solution should be stored at 4°C for a maximum of 1 month.
    ***Note:*** Each injection is performed using a new disposable needle. The tooth extraction surgery schedule to be carried out at the weekend of the 6^th^ week after the first drug injection.
2. For ORNJ: Mouse anesthesia.
  **Timing: 10 min**
  ***Note:*** This section describes the process of animal anesthesia.
  a. House mice (8–12 weeks old male C57BL/6J) in standard cages with a 12 h light/dark cycle and ad libitum access to food and water.
  b. Weigh each mouse. Inject the mice intraperitoneally with the prepared solution at a dose of 0.2 mL/10g of tribromoethyl alcohol (Avertin).
3. For ORNJ: Mouse positioning.
  **Timing: 5 min**
  ***Note:*** This section describes how to fix the mouse in a customized lead chamber, which enables precise radiation exposure of mandible.
  a. Place the mouse in the customized lead chamber in a supine position (Figure 1A).

**Figure 1:**
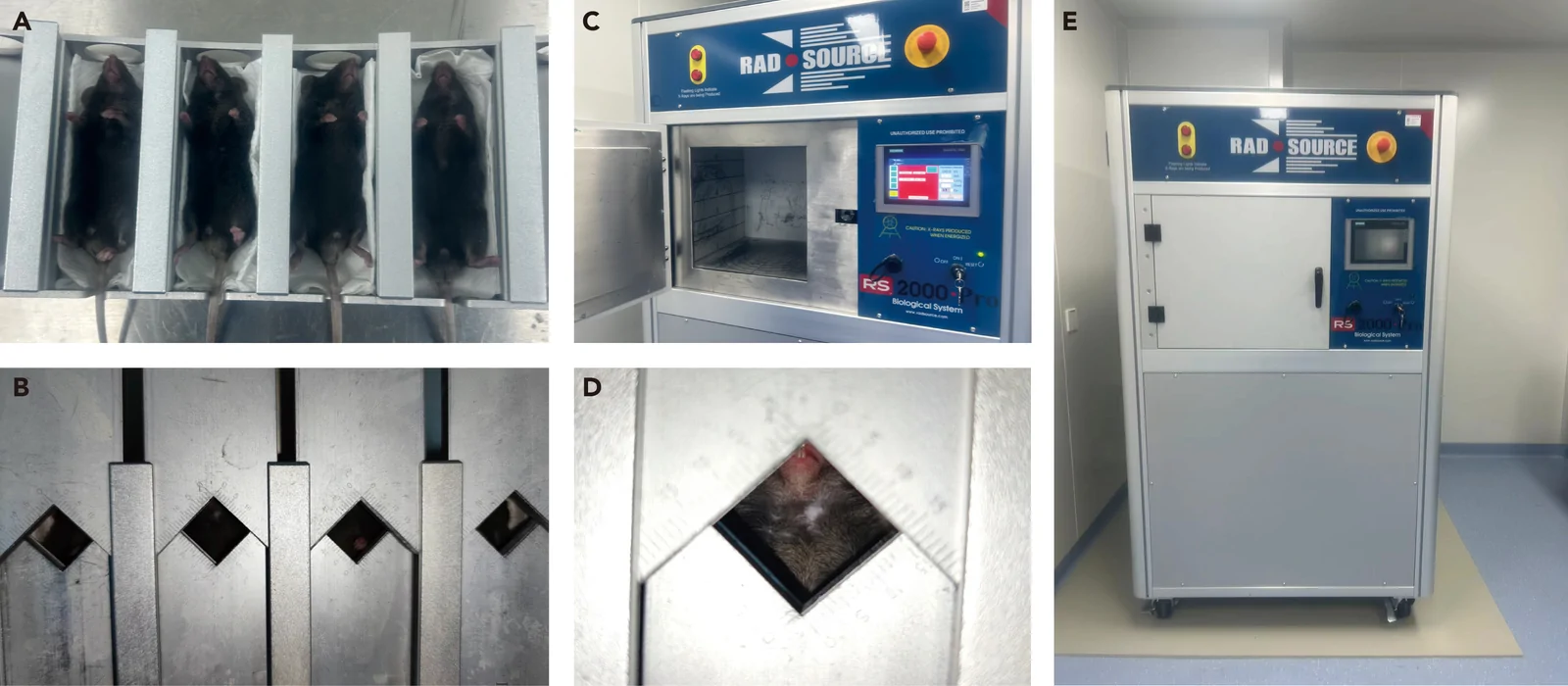
The establishment and validation of the mouse ORNJ model (A) Placement of the mouse in the customized lead chamber. (B) Close the lid to protect the mice from excess radiation injury. (C) The radiation cabin which accommodates four mice. (D) The gap on the lid for exposing the area of mouse mandible. (E) Set the radiation dose and start the irradiator.
  b. Close the lid and leave a square opening to expose only the mandible.
  c. Adjust the area of the gap according to the size of the mandible (Figures 1B–1D). Specifically, set the mandibular incisors as the upper boundary of the irradiation field, and touch the inferior border of mandible to determine the lower boundary of the irradiation field. The chamber can accommodate 4 mice at a time.
4. For ORNJ: Radiation treatment.
  **Timing: 6 min**
  ***Note:*** This section describes how to administer radiation exposure to the mice.
  a. Set the single radiation dose to 16 Gy and start radiation (Figure 1E).
  b. Take out the mice from the chamber and transfer them to the cage.
    ***Note:*** Leave the room where the X-ray irradiator is located to avoid the impacts of radiation on operators. Set sham-irradiated control group, in which the mice are placed in the chamber after anesthesia, but without radiation exposure.
5. For ORNJ: Post-radiation care.
  **Timing: 2 weeks**
  ***Note:*** This section describes the managements after radiation.
  a. Feed the mice in standard cages with a 12 h light/dark cycle and ad libitum access to food and water.

### Surgical instrument preparation

**Timing: 20 min (excluding the time for sterilization)**

This section describes how to prepare the necessary equipment for an extraction procedure, which is carried out at the end of the 6th week of drug injection.

6. Prepare the following instruments in advance before the tooth extraction surgery and place them in the high-pressure sterilization and disinfection surgical instruments:
  a. micro-forceps, the tip of which should be bent beforehand (Figure 2B), to grasp the bifurcation when extracting the mandibular first molar (M1) and loosen the tooth before extraction.

**Figure 2:**
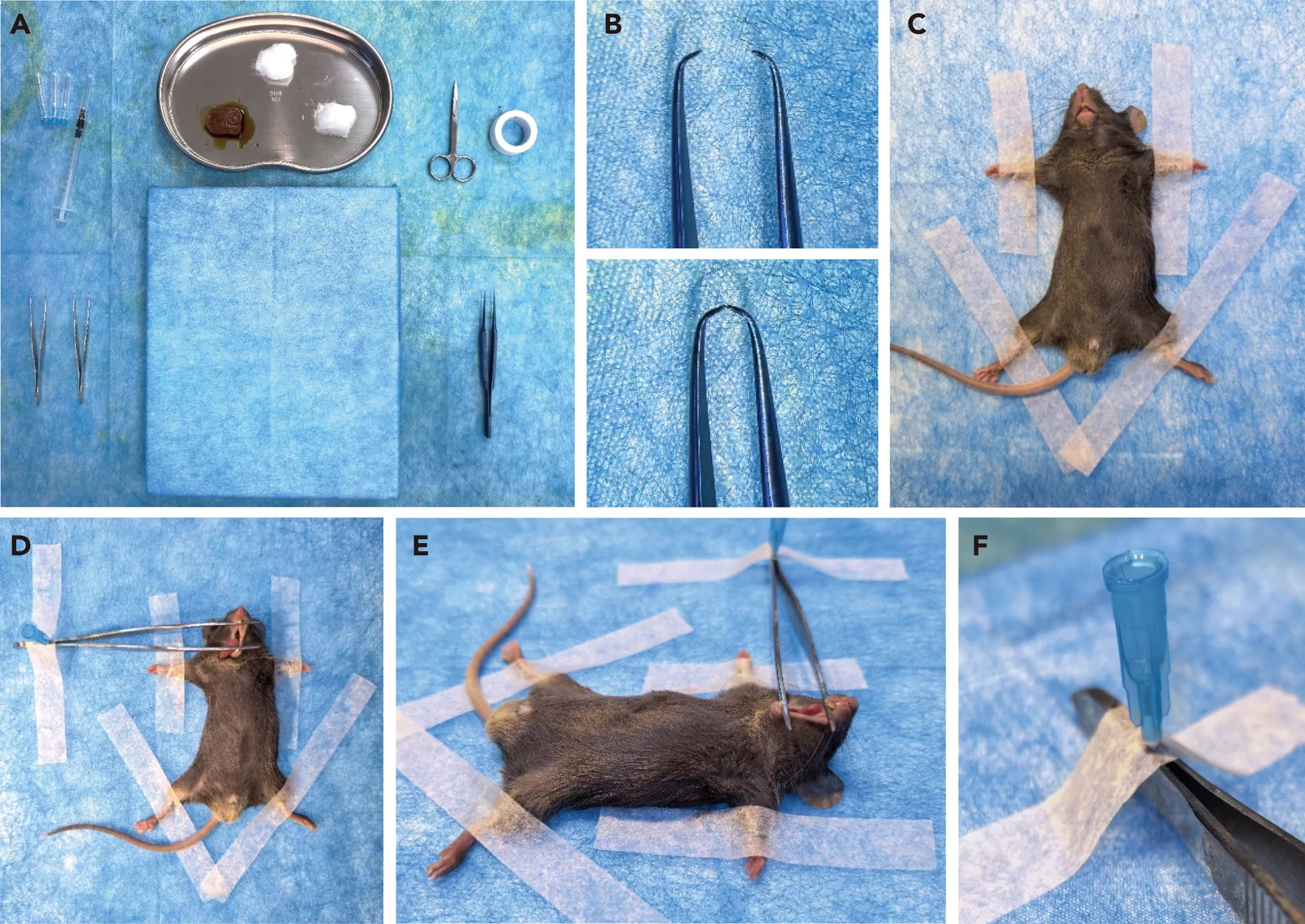
The surgical preparation and positioning of the mouse before tooth extraction (A) Complete surgical platform and required instruments. (B) The tip of the bent micro-forceps. (C–F) Methods for immobilizing mice and opening the mouse’s mouth.
  b. Blunt tissue forceps, one of which used as a buccal retractor when exposing the mandibular molar area and another used as a mouth opener (Figure 2D).
  c. Disposable syringe needles (at least 2 per mouse) to fix the mouth opener and separate the gingiva.
  d. Sterile cotton balls used for stopping bleeding and cleaning the surgical site (Figure 2A).
  e. Ophthalmic scissors to cut tape.

***Note:*** Use a high-pressure sterilizer to perform a 15-minute sterilization process at a temperature of 121°C to disinfect all surgical instruments and materials.

7. Use disposable surgical drapes to set up the surgical table for mice. Disinfect the surgical table (a foam board, 32×22×2.5cm), injection syringe, tape and heating pad (37 °C, 18×10 cm) with 75% ethanol.

***Note:*** Disinfection tape is used to fix the limbs of the mice and the open forceps. A disinfection heating pad is used to maintain the body temperature of the mice during intraoperative and postoperative recovery phases.

8. Prepare the sterile surgical area.
  a. Disinfect the surgical table with 75% ethanol.
  b. Put on the sterile disposable surgical gown, gloves, mask, and head cover.
  c. Place the sterilized instruments and materials that have been wiped with 75% ethanol on the disinfected surgical table.
  d. Tear sterile cotton balls into small pieces with a diameter of about 5 millimeters for better use.

***Note:*** Before starting the aseptic procedure, make sure that the lighting in the surgical area is sufficient. Using a small table lamp or a head-mounted lamp is usually the common method.

### Animal preparation

**Timing: 10–15 min per mouse**

This section describes how to administer anesthesia to the mice, how to fix the mice and open their mouths to expose the surgical area.

9. Anesthetize the mice intraperitoneally.
  a. Weigh the mice.
  b. Fix the mouse with its abdomen facing upwards.
  c. Insert the needle about 1/3 away from the abdominal white line.
  d. After the needle tip penetrates the skin, tilt it slightly and insert it into the abdominal cavity and slowly inject a dose of 0.2 mL/10g Avertin.
  e. Confirm adequate anesthesia by the absence of pedal reflex (toe-pinch).

***Note:*** During the entire surgical procedure, monitor the depth of anesthesia continuously to ensure no reflex reactions occur, especially when extracting a tooth. Maintain body temperature with the heating pad throughout the procedure.

10. Apply a thin layer of sterile, preservative-free sodium hyaluronate eye drops (0.1%; e.g., Runyi®, Qilu Pharmaceutical, NMPA H20133263) to both corneas immediately after anesthetic induction.

**CRITICAL:** Eye drops are used to prevent corneal drying during the 30–60 min procedure; reapply if surgery extends beyond 45 min.

11. Position the mouse.
  a. Place the mouse on the surgical table and position it in a supine position.
  b. Secure the limbs with tape.
  c. Use one of the tissue forceps to open the mouth through the upper and lower incisors, and fix the tissue forceps to the surgical table by the disposable syringe needles and the tape (Figures 2C–2F).

***Note:*** Ensure that the line connecting the upper and lower incisors is parallel to the horizontal plane. The head of the mouse should be directed to the right side so as to facilitate the extraction of the lower molars.

### Tooth extraction

**Timing: 5–10 min per mouse**

This section describes how to use the homemade micro-forceps to remove the mandibular M1 from a mouse. Extraction of healthy mandibular M1 with intact periodontium is used to exclude pre-existing infection-related confounders, allowing isolated assessment of medication/radiation-induced pathology.

12. Expose the surgical area.
  a. Use blunt-tipped forceps to gently open the mouth and expose the area of the left lower molars (Figures 3A and 3B).

**Figure 3:**
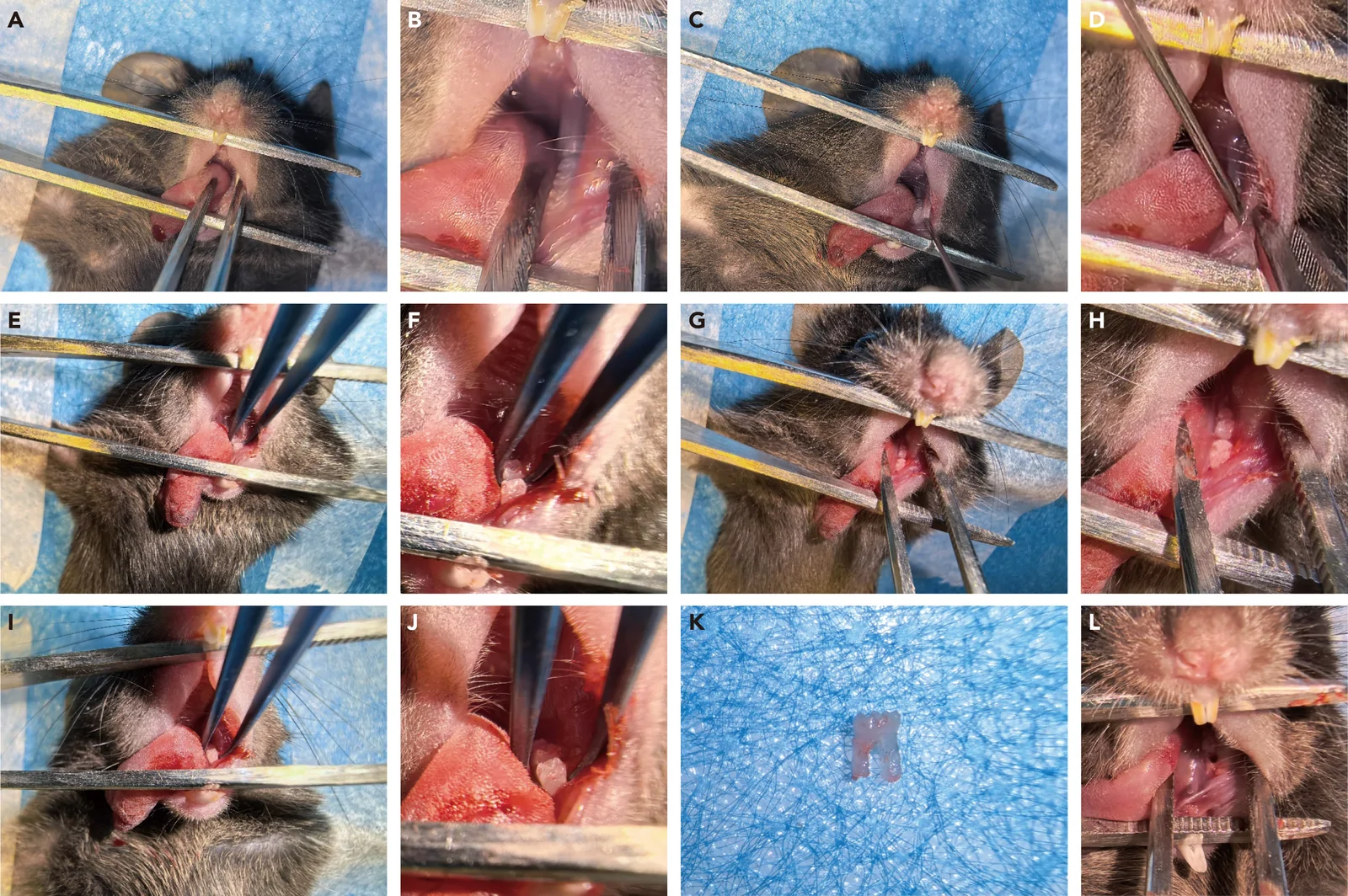
The detailed steps of tooth extraction (A and B) Expose the tooth extraction site. (C and D) Use a disposable syringe to separate the gingiva. (E and F) Use the tip of the micro-forceps to loosen left M1. (G and H) There is a gap between the two molars. (I and J) Remove teeth. (K and L) The extracted M1 tooth and the socket in the oral cavity for tooth extraction.
  b. Illuminate the surgical field using a focusable yellow LED lamp (3–5 W). Position the light source 10–15 cm from the maxillofacial region at a 60–80° incident angle relative to the operative plane.

***Note:*** This configuration maximizes contrast of the gingival vasculature and tooth morphology against the pink oral mucosa while minimizing glare and thermal buildup.

13. Use sterile cotton balls with normal saline to clean the tooth surfaces and gum grooves.
14. Use sterile cotton balls with povidone-iodine solution to gently wipe the left M1 area for about 5–10 s.

***Note:*** Use small cotton balls that are only slightly moist and do not drip to pour or squeeze. If necessary, use dry cotton balls to remove any excess residual liquid. Be extremely careful to prevent the liquid from flowing into the throat.

15. Pull the left buccal mucosa of the mouse. Use disposable syringe needles to separate the gingiva of left M1 (Figures 3C and 3D).

***Note:*** Be gentle with the movements to avoid injury to blood vessels and causing excessive bleeding.

16. Close the tips of the bent micro-forceps to pass through the gap between the first and second molars on the lower jaw (Figures 3E and 3F). Rotate along the long axis of the instrument to loosen left M1.

***Note:*** Avoid applying too much force during the clamping process to prevent damaging the tooth crown. Loosen the crown of left M1 towards the buccal side firstly, and then loosen it towards the labial side.

17. Pull out left M1.
  a. Use the bent micro-forceps to firmly grasp the root bifurcation of left M1, apply force along the long axis of the tooth, and perform lateral movements in both the buccal and palatal directions (Figures 3I and 3J).
  b. During the shaking process, apply vertical force towards the occlusal surface to dislodge the tooth and complete the extraction.

***Note:*** When shaking, carefully feel the degree of looseness between the alveolar bone and the teeth. The shaking should be gradually intensified. When there is no obvious resistance, try to apply force vertically towards the occlusal surface.

**CRITICAL:** The force applied during clamping should not be too strong to avoid crushing the dental crown. Do not reach into the soft tissues to avoid causing bleeding.

18. Check whether the tooth root is intact, and whether there are any fragments of alveolar bone or large masses of soft tissue attached to it.
19. Clean the surgical area with normal saline.
20. Remove the right M1 similarly following the above steps.

***Note:*** Each mouse needs to have all the instruments disinfected before the tooth extraction surgery.

### Postoperative care

**Timing: 1–2 h per mouse**

This section describes the postoperative care for BRONJ and ORNJ after the tooth extraction.

21. Remove the mouse gently from the fixed surgical plate after the surgery.
22. Drop a drop of normal saline on the tip of the mouse’s tongue to moisten its mouth.

***Note:*** The amount of normal saline dropped should not be excessive, as excessive liquid may enter the respiratory tract, causing the mouse to cough and suffocate.

23. Place the mouse on the heating pad and keep it in a prone position.

***Note:*** The head should be tilted to one side to prevent liquid from entering the mouse's respiratory tract.

24. Maintain this position until the mouse regains consciousness. Continuously observe whether the mouse has regained consciousness, whether there is breathing difficulty, and whether there is continuous bleeding in the surgical area.

***Note:*** This process usually lasts for 1–2 h. During this period, a drop of normal saline can be applied to the tip of the mouse's tongue every half an hour.

25. Transfer the animals to clean cages and feed the mice in standard cages with a 12 h light/dark cycle and ad libitum access to food and water.
  a. Provide fresh and softened food.
  b. Administer buprenorphine (0.05–0.1 mg/kg SC) immediately after anesthetic recovery, repeated every 8–12 h for 72 h.

### For BRONJ: Postoperative drug injection

**Timing: 2 weeks**

This section describes the postoperative drug injection only for BRONJ.

26. After the surgery, administer ZA continuously for two more weeks at a dose of 200 μg/kg, 3 times a week.

***Note:*** Continuously monitor whether the mice show signs of swelling, bleeding, infection or pain. If any severe cases are found, euthanasia should be carried out immediately.

### For ORNJ: Postoperative management

**Timing: 2 weeks**

This section describes the postoperative management only for ORNJ.

27. After the surgery, feed the mice in standard cages with a 12 h light/dark cycle and ad libitum access to food and water.

### Tissue collection

**Timing: 15–20 min**

This section describes the steps for tissue collection, which is two weeks after the tooth extraction.

28. After the mice complete 8 weeks of drug injection, euthanize the mouse using carbon dioxide (CO_2_) inhalation until they stopped moving. Use cervical dislocation to ensure the mice totally death.
29. Collect the mandibles of the mice.
  a. Open the skin and muscles on the cheek of the mice, exposing the bone surface.
  b. Cut off the mucosa and muscles attached to the inner side of the mandible from the mouth of the mice.
  c. Separate the condyle of the temporomandibular joint carefully.
  d. Gently remove the soft tissues on the bone surface using scissors and forceps.

***Note:*** Be careful not to damage the new bone tissue in the extraction socket.

30. Immerse the mandibles of the mice in 4% PFA solution at 4°C for 3 h.

**CRITICAL:** PFA is highly toxic. Store the solution at 4 °C for up to 1 weeks and prepare fresh for best results.

31. Rinse 3 times with PBS for 5 min each.
32. Store the samples in PBS at 4°C.

### Micro-CT analysis

**Timing: 1–2 h per sample for micro-CT; 20–30 min per sample for morphometric analysis**

This section describes the steps for micro-CT analysis.

33. Remove the mandibles from the PBS and place it in the micro-CT scanner with preset parameters.

***Note:*** This protocol uses a 34mm diameter sample tube for scanning, with Voxel size 10.0 μm, FOV 35.36 mm, Image matrix 3400×3400×775, Slices 775, Voltage 55 kVp, Intensity 145 μA for reference.

34. Return the scanned samples in PBS for storage.
35. Reconstruct 3D images using the manufacturer’s software (DataViewer and CTvox). Set lower and upper gray threshold values to 130 and 255.

***Note:*** The region of interest (ROI) is defined as the extraction socket of the first molar on the mandible.

### Decalcification

**Timing: 5 days**

This section describes the steps for decalcification before histology or immunofluorescence analysis.

36. Decalcify in 0.5 M EDTA (pH 7.4) for 5 days at 20–25°C, changing the solution every day.

***Note:*** Store EDTA solution at 20–25°C for up to 1 month.

37. Wash the samples 3 times using PBS for 10 min each.

***Note:*** The objective is to render the mandible pliable for intact 3μm sectioning, not absolute demineralization (which compromises antigenicity). Use samples only scanned by micro-CT or new ones. The method to confirm the mandible has been demineralized is to insert a disposable syringe needle into the thicker part of samples. If the needle can be inserted easily, decalcification is successfully achieved.

### After decalcification: Histology

**Timing: 1 day**

This section describes histology after decalcification.

38. Process the samples by the automatic tissue processor treated with ethanol of different concentrations (70%, 80%, 95% and 100%) first and then xylene.
39. Insert the samples into the embedding mold and confirm that the sagittal plane is correct.
40. Quickly inject 60 °C paraffin and gently press the specimen to prevent air bubbles. Cool at 20–25°C until complete solidification.

***Note:*** The longitudinal axis of the molar row is usually the key observation plane for the sagittal section. Store at 4 °C for a short period for easy slicing.

41. Section and attach the section to a positively charged adhesive slide. Use H&E staining and TRAP Staining to evaluate the modeling effects of the model of BRONJ in mice.

***Note:*** Osteoclasts are defined as multinucleated (≥3 nuclei) TRAP-positive cells lining the bone surface, a standard criterion for ORNJ evaluation. The thickness of the section is 3 μm in this step.

42. Place and scan in the digital slide scanner.

### After decalcification: Immunofluorescence analysis

**Timing: 3 days**

This section describes immunofluorescence analysis after decalcification.

43. Subject the samples to dehydration treatment with 20% sucrose and 2% polyvinylpyrrolidone in 4 °C for 24 h.
44. Embed the samples 20% sucrose, 2% polyvinylpyrrolidone and 8% gelatin. Place embedded samples in a −20 °C freezer for 45 min.

***Note:*** Place samples in the freezer to facilitate faster solidification.

45. Cut the samples into sections using a low-profile blade on the cryostat and dry in air before being stored in a freezer in −20 °C.

***Note:*** Unlike conventional FFPE processing which often requires heat-induced epitope retrieval (HIER) due to extensive cross-linking and mineral chelation, our cryoprotection strategy stabilizes protein conformations during the mild 4°C EDTA decalcification. Therefore, antigen retrieval steps (e.g., citrate buffer heating) are omitted to prevent epitope denaturation and tissue loss, ensuring optimal signal-to-noise ratios for high-resolution 3D confocal microscopy. The thickness of the section is 80 μm in this step.

46. Perform permeabilization with Triton X-100, block with 5% donkey serum.
47. Conduct immunostaining with antibody solution. Add the nuclear marker DAPI to the secondary antibody solution at a ratio of 1:1000 for staining. Include parallel assay controls to validate staining specificity:
  a. Positive tissue controls: adjacent sections of mouse spleen (for CD31 and Endomucin) and calvarial growth plate (for Sp7);
  b. Negative antibody controls: isotype-matched IgG replacing primary antibodies.

**CRITICAL:** The antibody solution should be stored at 4°C in a place from which all such light is excluded for a maximum of 1 weeks. Prepare fresh for best results.

***Note:*** Add CD31, Endomucin, Sp7 to the primary antibody solution at a ratio of 1:200 for staining. Add Donkey anti-Mouse IgG Alexa Fluor 488, Donkey anti-Goat IgG Alexa Fluor 555, Donkey anti-Rabbit IgG Alexa Fluor 647 to the primary antibody solution at a ratio of 1:500 for staining. All primary antibodies are validated for use on EDTA-decalcified murine bone in established workflows: CD31 and Endomucin in this protocol analogous to Kusumbe et al.^15^; Sp7 as in Xie et al.,^17^ who employed identical 4% PFA fixation and EDTA decalcification on murine mandibles.

48. Place and scan in the spinning disk confocal microscope.
49. Process data.
  a. Capture the images on the digital slide scanner are processed using OlyVIA.
  b. Use Imaris and Imaris File Converter to analyze the images captured on the spinning disk confocal microscope.
    i. Use Imaris for 3D quantitative analysis of Sp7 distribution.
    ii. Acquire high-resolution Z-stack confocal images (step size: 0.5–1 μm) of the immunofluorescently labeled mandibles.
    iii. Import raw image files into Imaris for 3D reconstruction and quantitative analysis.
50. Use Adobe Illustrator software for image processing and analysis.
  ***Note:*** To quantify osteogenic activity, utilize the “Surfaces” creation algorithm to render Sp7^+^ (osterix) nuclei (identified by DAPI co-localization) within the region of interest (ROI; e.g., the extraction socket). Define and export the following quantitative endpoints:
  a. **Sp7**^**+**^ **Cell Density:** Quantified as the total number of Sp7^+^ cells per mm^2^ of bone surface area.
  b. **Sp7**^**+**^ **Volume Fraction:** Calculated as the total volume occupied by Sp7^+^ cells divided by the total volume of the ROI.
  c. **Spatial Distribution:** Utilize the “Distance Transformation” tool to analyze the spatial relationship between Sp7^+^ cells and the bone surface or vasculature (CD31^+^/Endomucin^+^), providing metrics on osteoblast localization.
    ***Note:*** These standardized Imaris workflows provide robust, reproducible computational endpoints for assessing altered osteogenesis in downstream mechanistic studies.

## Expected outcomes

Using this protocol, mice are expected to develop consistent and reproducible BRONJ within 8 weeks and ORNJ within 4 weeks. Compared with control mice (Figures 4A and 4B), BRONJ mice typically exhibit delayed or incomplete mucosal healing with partial bone exposure at the extraction site (Figures 4D and 4E). Micro-CT and histological analyses further demonstrate impaired bone regeneration in the extraction socket with the presence of necrotic bone tissue (Figure 5A). Immunolabeling reveals reduced endothelial cell abundance and decreased osteogenic activity within BRONJ lesions (Figure 5C). In ORNJ mice, histological analysis demonstrates endothelial swelling, microthrombus formation, and enlarged bone lacunae (Figure 5B), consistent with radiation-induced bone damage and impaired bone remodeling. Notably, ORNJ lesions exhibit increased numbers of multinucleated cells adjacent to bone surfaces; definitive quantification of increased osteoclast activity should be confirmed via mandatory TRAP staining, as morphological assessment alone may be inconclusive.

**Figure 4:**
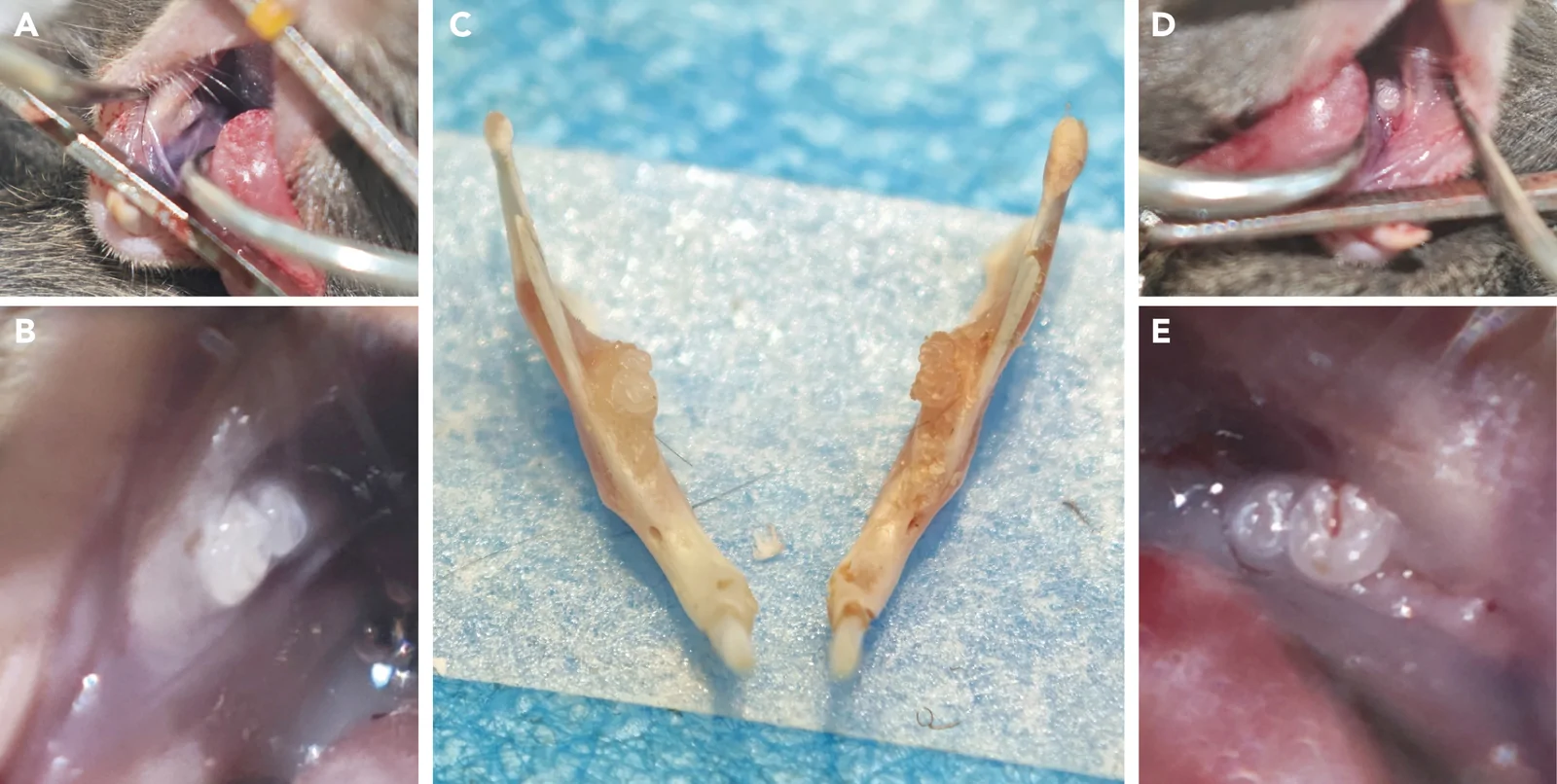
The comparison between samples of BRONJ and control (A and B) Intraoral images of control. (C) Comparison of samples between the BRONJ group and the control group, with. (D and E) Intraoral images of BRONJ shows poor healing of the oral mucosa.

**Figure 5:**
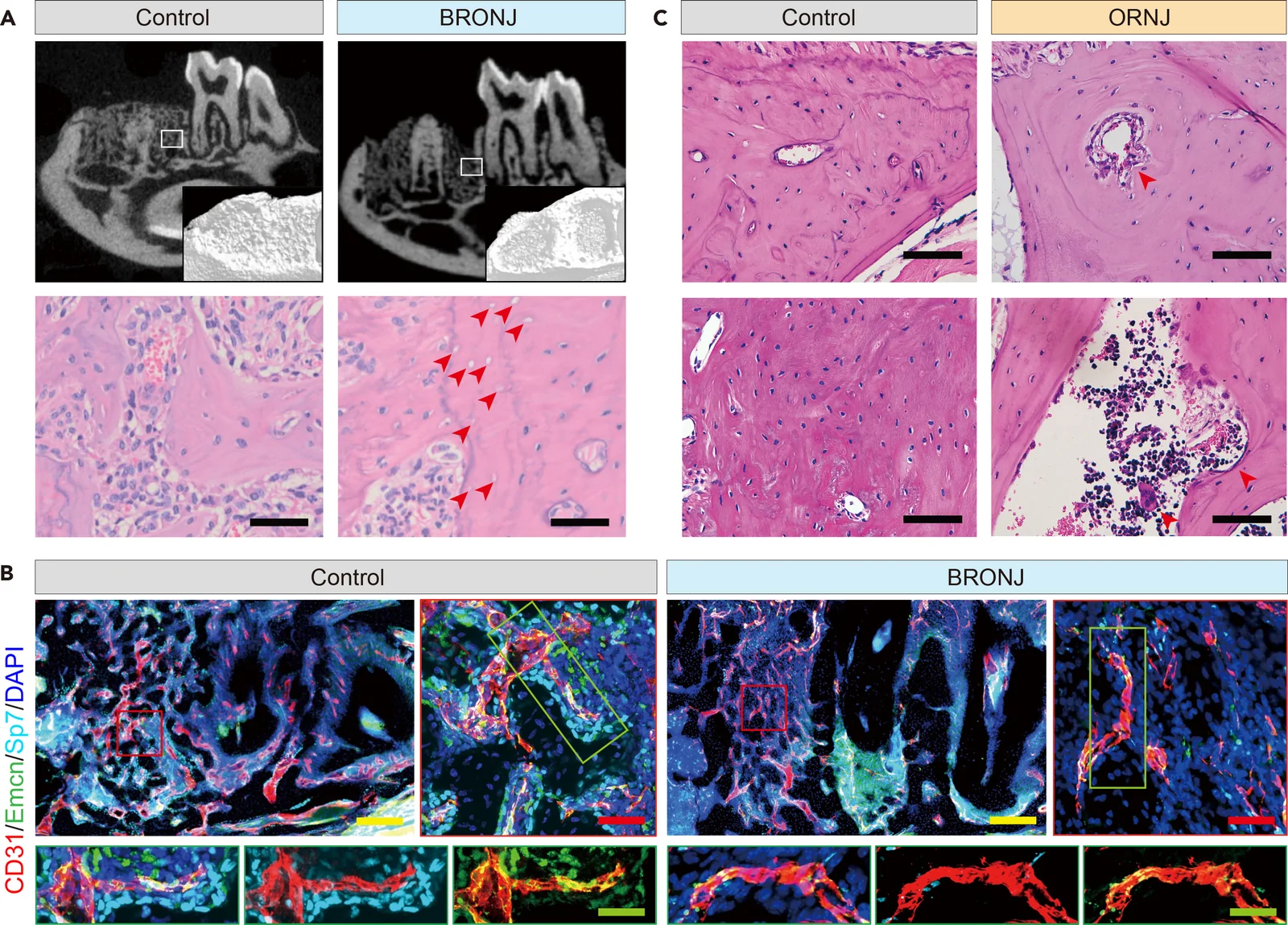
Analysis of the BRONJ and ORNJ compared with control (A) Micro-CT shows lower bone density in BRONJ. Scale bars: 500 μm. H&E staining shows that pathological manifestations of bone lacuna. Scale bars: 50 μm. (B) The H&E staining of mandible sample sections shows that pathological manifestations of vasculature, osteoclast, and bone lacuna. Scale bars: 100 μm. (C) Immunolabeling in BRONJ shows reduced endothelial cell abundance and decreased osteogenic activity. Scale bars: 100 μm (yellow), 40 μm (red), 15 μm (green).

## Limitations

This protocol provides practical and reproducible steps for establishing mouse BRONJ and ORNJ models; however, several limitations should be considered. First, although detailed procedures for tooth extraction are provided, the surgery still requires advanced microsurgical skills. Inexperienced operators may encounter a higher failure rate, as improper manipulation can cause excessive trauma or even mandibular fracture, thereby compromising experimental outcomes. Second, this protocol is optimized for young adult mice. In older mice, particularly those aged over 50 weeks, age-related alterations in bone architecture may increase the risk of root fracture during molar extraction. Notably, BRONJ lesions can still develop even when residual roots remain. Third, the protocol exclusively uses male mice. While this avoids estrous-cycle confounds and enhances reproducibility, it limits translational relevance: clinical BRONJ predominantly affects postmenopausal women on bisphosphonates, wherein estrogen deficiency impairs healing. Although ORNJ shows less sex bias, radiation responses may differ by sex. Future studies should consider ovariectomized females to model the clinical at-risk population. Fourth, ZA administration alone does not reflect the clinical reality that most MRONJ cases occur in patients receiving antiresorptives alongside additional risk factors including systemic glucocorticoids (e.g., dexamethasone), chemotherapy, or anti-angiogenic agents, a nuance our initial description understated. To improve translational fidelity, future studies should adopt the widely used “ZA + dexamethasone” combination regimen. We are also currently integrating this model with multi-omic analyses of clinical drug-induced jaw necrosis cohorts to better align preclinical findings with patient heterogeneity. Fifth, a single high-dose irradiation regimen is employed to induce injury, which differs from the fractionated radiotherapy used in clinical practice. Although this approach may increase mortality due to extensive tissue damage, it enables more rapid induction of ORNJ. Sixth, the protocol uses extraction of healthy teeth with intact periodontal tissues. While this minimizes inflammatory confounds to isolate ZA/radiation toxicity, it does not reflect the clinical scenario where BRONJ/ORNJ frequently develops after extraction of infected teeth. Future studies should combine localized periodontal pathogen challenge to better recapitulate clinical disease drivers. Despite these limitations, the protocol provides a robust and controllable platform for preclinical studies of jaw osteonecrosis.

While this protocol provides a robust murine model for BRONJ/ORNJ, it is essential to distinguish its utility from other established systems. Recent large-animal work, such as Göttingen minipig BRONJ models with macroscopic/radiological/histological scoring schemes demonstrates infection-triggered MRONJ without surgical intervention and underscores the value of porcine systems for anatomical homology and late-stage pre-clinical validation.^18^ Concurrently, international consensus efforts such as the 2022 AAOMS position paper^19^ and the 2024 Italy MRONJ Symposium collating AEIRS, North American, and European expert perspectives^20^) have further standardized endpoint definitions and imaging criteria across consortia. In contrast, the present murine model is optimized for high-throughput mechanistic screening. It leverages high-resolution microCT for detailed trabecular morphometry and a streamlined immunofluorescence workflow (quantifying CD31, Endomucin, and Sp7 via Imaris, as detailed in Step 38), anchored on antibodies validated in EDTA-decalcified murine bone.^15^^,^^17^ These analytical pipelines generate robust, reproducible quantitative endpoints that are logistically challenging and cost-prohibitive to scale in porcine systems, a limitation also reflected in recent systematic reviews of rodent MRONJ models.^21^^,^^22^ We therefore propose a translational pipeline: murine models for target discovery and proof-of-concept, followed by porcine or consortium-standardized models for late-stage validation. This positions our protocol as a complementary upstream platform within the broader pre-clinical MRONJ landscape.

## Troubleshooting

### Problem 1

Inadequate radiation injury (step “preoperative management”, 4).

### Potential solution

Ensure sufficient anesthesia to avoid changed location of mouse mandible. Adjust the chamber lid to expose the mandible to radiation area.

### Problem 2

Inadequate anesthesia (step “animal preparation”, 9).

### Potential solution

Inject the reagents strictly and slowly according to the dosage provided by the reagent manufacturer and the actual weight of the mice. Pay attention to the position of the mouse during the injection and the depth of the needle insertion. Do not make large movements that may cause bleeding in the abdominal cavity of the mouse.

Continuously monitor whether the reflexes of the mice exist (such as toe-pinch and righting reflex). If reflexes are observed, promptly replenish the dosage according to the instructions provided by the reagent manufacturer.

### Problem 3

Excessive bleeding during surgery (step “tooth extraction”, 15–17).

### Potential solution

During the operation, be careful not to damage the soft tissues such as the gums, tongue, and mucosa with sharp metal instruments. The forceps used to open the mouth can be separated by several cotton balls and the mouse’s tongue to prevent mechanical damage. Be careful to insert the micro-forceps into the root bifurcation area gently and do not clamp the gums.

Prepare a certain number of dry cotton balls in advance. In case of accidental puncturing of blood vessels and bleeding during the surgery, these cotton balls can be used to promptly absorb the blood and immediately disinfect with povidone-iodine cotton balls. Pre-position several cotton balls in the oral cavity. If the operation is proficient, be careful not to affect the breathing of the mouse.

### Problem 4

Crown fracture (step “tooth extraction”, 17).

### Potential solution

Avoid using sudden and excessive external force. Instead, apply controlled and gradual force. Find a stable fulcrum and you can appropriately generate force through the movements of your wrist and arm.

For Crown fracture, try to loosen the tooth root from the alveolar bone using a needle, and use a micro-forceps for a precise extraction. It is recommended to practice on a mouse cadaver before the actual surgery.

### Problem 5

Weight loss (step “postoperative care”, 25).

### Potential solution

Provide appropriate soft food. Adhere to the prescribed buprenorphine regimen. Monitor for >15% weight loss or distress; apply IACUC-approved humane endpoints if unresponsive.

### Problem 6

Death after radiation and tooth extraction (step “preoperative management”, 5; “postoperative care”, 25).

### Potential solution

Avoid excessive exposure to radiation and additional injury. Check the setting of irradiator to ensure proper radiation dose. Provide softened food after radiation and tooth extraction.

## Resource availability

### Lead contact

Further information and requests for resources and reagents should be directed to and will be fulfilled by the lead contact, Anjali P Kusumbe (anjali.pkusumbe@ntu.edu.sg).

### Technical contact

Questions about the technical specifics of performing the protocol should be directed to the technical contact, Junyu Chen (junyuchen@scu.edu.cn).

### Materials availability

This study did not generate new unique reagents.

### Data and code availability

This study did not generate or analyze datasets. This study did not report original code.

## Acknowledgments

J.C. is supported by 10.13039/501100001809National Natural Science Foundation of China (nos. 82422021 and 82270961), 10.13039/501100020207Sichuan Provincial Health Commission (24QNMP015), and West China Hospital of Stomatology Sichuan University (RD-03-202401 and RCDWJS2024-5). A.P.K. is supported by 10.13039/501100001459Ministry of Education (MOE) Singapore: Academic Research Funds (nos. 024983-00001 and 025277-00026), European Research Council (StG: metaNiche, 805201), and European Union’s Horizon 2020 (no 857524). A.C. is supported by the 10.13039/100000002National Institutes of Health (NIH) under grant R01AG082681. M.V.R. acknowledges support by the 10.13039/100000049National Institute on Aging (R01AG073349) and 10.13039/100000069National Institute of Arthritis and Musculoskeletal and Skin Diseases (R01AR055655 and R01AR082460).

## Author contributions

Z.D., J.Z., and H.L., methodology, investigation, visualization, and writing. A.C. and M.V.R., reviewing and editing. J.C., methodology, validation, and formal analysis. A.P.K., supervision, reviewing, and editing.

## Declaration of interests

The authors declare no competing interests.
